# Cancer diagnosis and prognosis decoded by blood-based circulating microRNA signatures

**DOI:** 10.3389/fgene.2013.00116

**Published:** 2013-06-21

**Authors:** Dharanija Madhavan, Katarina Cuk, Barbara Burwinkel, Rongxi Yang

**Affiliations:** ^1^Molecular Epidemiology, German Cancer Research CenterHeidelberg, Germany; ^2^Molecular Biology of Breast Cancer, Department of Gynecology and Obstetrics, University ClinicHeidelberg, Heidelberg, Germany

**Keywords:** circulating miRNA, cancer, biomarkers, prognostic marker, detection marker

## Abstract

In the recent years, circulating microRNAs (miRNAs) have garnered a lot of attention and interest in the field of disease biomarkers. With characteristics such as high stability, low cost, possibility of repeated sampling and minimal invasiveness, circulating miRNAs are ideal for development into diagnostic tests. There have been many studies reported on the potential of circulating miRNAs as early detection, prognostic, and predictive biomarkers in cancer. Here, we have reviewed the application of plasma and serum miRNAs as biomarkers for cancer focusing on epithelial carcinomas [prostate, breast, lung, colorectal, and gastric cancer (GC)] and hematological malignancies (leukemia and lymphoma). We have also addressed the common challenges that need to be overcome to achieve a successful bench to bedside transition.

## Introduction

Cancer accounts for the highest mortality in developed countries, and second highest in developing countries, making it a worldwide health problem (Ferlay et al., 2008; Siegel et al., 2012). One method of disease management is the development of biomarkers, which could enhance early cancer detection, improve patient stratification and therapy response prediction. This in turn would result in a more favorable disease outcome. Blood-based protein biomarkers, such as carcinoembryonic antigen (CEA), carbohydrate antigen (CA), or prostate specific antigen (PSA), have gained a lot of recognition. Nevertheless, they suffer from low sensitivity, especially with respect to their use in screening for early stages or inability to distinguish aggressive from indolent tumors (Lumachi et al., 2000; Hanash et al., 2011). This has prompted the investigation for novel and more sensitive biomarkers, which can supplement or complement existing detection methods and aid in disease monitoring.

**Figure 1:**
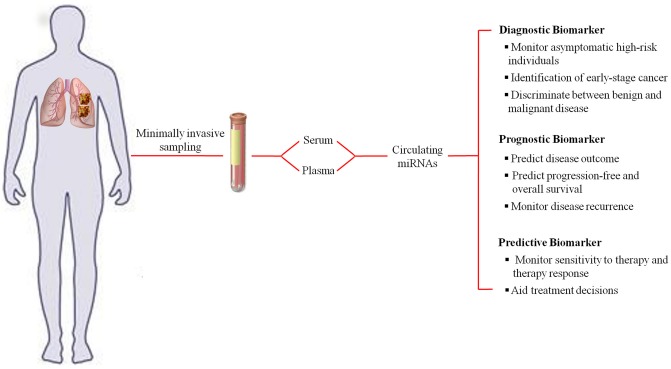
**Representation of the diagnostic, prognostic, and predictive uses of circulating miRNAs as a biomarker using lung cancer as an example**.

Since their discovery, microRNAs (miRNAs) have proven to be an essential and indispensable layer in gene regulation, mainly, post-transcriptional regulation. Involved in many physiological processes and also found to be deregulated in many disease mechanisms, miRNAs have also gained importance as they carry information about the patho-physiological state of a person, and thus can serve as biomarkers. miRNAs present in the cell-free body fluids such as plasma, serum, urine, saliva, etc., termed as circulating miRNAs, have been exploited as biomarkers in many diseases in the past five years. Placental miRNAs were the first class of circulating miRNAs to be detected in maternal plasma during pregnancy (Chim et al., 2008). Parallel to this discovery, miRNAs were found to be elevated in the serum of lymphoma patients compared to healthy individuals (Lawrie et al., 2008). Since then circulating miRNAs have attracted a great deal of attention as novel, minimally invasive biomarkers for various diseases. Features such as ease of access and remarkable stability increase their potential as disease biomarkers (Mitchell et al., 2008; Turchinovich et al., 2011). Since they are bound to proteins like argonaute (AGO) or high density lipoprotein, they are highly resistant to RNase activity, unlike mRNAs measured in gene signature markers (Turchinovich and Burwinkel, 2012). Detection of miRNAs by quantitative polymerase chain reaction (qPCR) has the advantages of being relatively inexpensive, sensitive to even low amounts due to amplification of signal, and robust. In these terms, they seem to have an edge over protein markers, which currently represent the most common circulating biomarker type. A further testament to their diverse and dynamic uses in a clinical settings is the various reports outlining use of circulating miRNAs as a diagnostic, prognostic and predictive marker (Figure 1). The impact of circulating miRNAs in the field of biomarkers has been unequivocal with more than 250 studies probing their use in cancer diagnosis and prognosis.

In this review, we have summarized the recent-findings in cancer related circulating miRNAs and presented them also in a tabular form, with a focus on epithelial cancers such as prostate (Table 1), breast (Table 2), lung (Table 3), colorectal (Table 4), and gastric (Table 5) cancer, and also touched upon lymphomas and leukemia (Table 6). We present a comprehensive summary of circulating miRNAs present in plasma or serum which are associated with diagnosis and/or prognosis of both primary and metastatic cancers, along with an appraisal of the translational implications of this field.

**Table 1:** **Circulating miRNAs reprted to be a biomarker for prostate cancer**.

**miRNA**	**Sample type**	**Type of biomarker**	**miRNA levels and clinical significance**	**References**
let-7c	Plasma	Diagnostic	Decreased in PC compared to BPH and healthy controls	Chen et al., 2012a,b
let-7e	Plasma	Diagnostic	Decreased in PC compared to BPH and healthy controls	Chen et al., 2012a,b
let-7i	Serum	Diagnostic	Increased in mPC compared to BPH; correlated to Gleason score;	Mahn et al., 2011
miR-20a	Plasma	Prognostic	Correlated to rumor stage and D'Amico scores	Shen et al., 2012
miR-21	Serum	Predictive	Increased in HRPC, especially in those resistant to docetaxel-based chemotherapy	Zhang et al., 2011
miR-21	Plasma	Prognostic	Correlated to CAPRA scores and D'Amico scores	Shen et al., 2012
miR-26a	Serum	Diagnostic	Increased in mPC compared to BPH; decreased in post-operative samples	Mahn et al., 2011
miR-30c	Plasma	Diagnostic	Decreased in PC compared to BPH and healthy controls	Chen et al., 2012a,b
miR-141	Serum	Diagnostic	Increased in mPC; correlate to PSA levels	Mitchell et al., 2008
miR-141	Serum	Diagnostic and prognostic	Increased in mPC compared to localized PC; correlation to Gleason score and lymph node status	Brase et al., 2011
miR-141	Plasma	Prognostic	Correlation to CTC counts, PSA and LDH	Gonzales et al., 2011
miR-141	Serum	Diagnostic and prognostic	Increased in CRPC compared to localized PC	Nguyen et al., 2012
miR-145	Plasma	Prognostic	Correlated to D'Amico scores	Shen et al., 2012
miR-195	Serum	Diagnostic	Increased in mPC compared to BPH; correlated to Gleason score; decrease in post-operative samples	Mahn et al., 2011
miR-221	Plasma	Prognostic	Correlated to D'Amico scores	Shen et al., 2012
miR-375	Serum	Diagnostic and prognostic	Increased in mPC compared to localized PC; correlation to Gleason score and lymph node status	Brase et al., 2011
miR-375	Serum	Diagnostic and prognostic	Increased in CRPC compared to localized PC	Nguyen et al., 2012
miR-378^*^	Serum	Diagnostic and prognostic	Increased in CRPC compared to localized PC	Nguyen et al., 2012
miR-409-3p	Serum	Diagnostic and prognostic	Decreased in CRPC compared to localized PC	Nguyen et al., 2012
miR-622	Plasma	Diagnostic	Increased in PC compared to BPH and healthy controls	Chen et al., 2012a,b
miR-1285	Plasma	Diagnostic	Increased in PC compared to BPH and healthy controls	Chen et al., 2012a,b

Abbreviations: BPH, benign prostatic hyperplasia; CTC, circulating tumour cells; HRPC, hormone-refractory prostate cancer; LDH, lactose dehydrogenase; PC, prostate cancer; PSA, prostate specific antigen; mPC, metastatic prostate cancer.

**Table 2:** **Circulating miRNAs reprted to be a biomarker for breast cancer**.

**miRNA**	**Sample type**	**Type of biomarker**	**miRNA levels and clinical significance**	**References**
miR-10b	Serum	Diagnostic and prognostic	Increased in MBC compared to PBC and controls	Roth et al., 2010
miR-10b	Plasma	Prognostic	Increased in MBC	Chen et al., 2012a,b
miR-10b	Serum	Diagnostic	Increased in PBC	Mar-Aguilar et al., 2013
miR-21	Serum	Diagnostic	Increased in PBC; correlated to tumour size and lymph node status	Si et al., 2013
miR-21	Serum	Diagnostic	Increased in PBC	Mar-Aguilar et al., 2013
miR-30a	Plasma	Diagnostic	Decreased in PBC; correlated to receptor status	Zeng et al., 2013
miR-34a	Serum	Diagnostic and prognostic	Increased in MBC compared to PBC and controls	Roth et al., 2010
miR-92a	Serum	Diagnostic	Decreased in PBC; correlated to tumour size and lymph node status	Si et al., 2013
miR-125b	Serum	Predictive	Increased in patients not responding neoadjuvant chemotherapy	Wang et al., 2012a,b,c
miR-125b	Serum	Diagnostic	Increased in PBC	Mar-Aguilar et al., 2013
miR-141	Plasma	Diagnostic and prognostic	Increased in MBC; correlation to CTC status	Madhavan et al., 2012
miR-145	Serum	Diagnostic	Increased in PBC	Mar-Aguilar et al., 2013
miR-145	Plasma	Diagnostic	Decreased in PBC	Ng et al., 2013
miR-148b	Plasma	Diagnostic	Increased	Cuk et al., 2013
miR-155	Serum	Diagnostic	Correlation to progesterone receptor status	Zhu et al., 2009
miR-155	Serum	Diagnostic and prognostic	Increased in PBC and MBC compared to controls; increased in PBC compared to MBC	Roth et al., 2010
miR-155	Serum	Diagnostic and predictive	Increased in PBC; decrease after surgery and chemotherapy	Sun et al., 2012
miR-155	Serum	Diagnostic	Increased in PBC	Mar-Aguilar et al., 2013
miR-191	Serum	Diagnostic	Increased in PBC	Mar-Aguilar et al., 2013
miR-200a	Plasma	Diagnostic and prognostic	Increased in MBC; correlation to CTC status	Madhavan et al., 2012
miR-200b	Plasma	Diagnostic and prognostic	Increased in MBC; correlation to CTC status	Madhavan et al., 2012
miR-200c	Plasma	Diagnostic and prognostic	Increased in MBC; correlation to CTC status	Madhavan et al., 2012
miR-203	Plasma	Diagnostic and prognostic	Increased in MBC; correlation to CTC status	Madhavan et al., 2012
miR-210	Plasma	Diagnostic and prognostic	Increased in MBC; correlation to CTC status	Madhavan et al., 2012
miR-215	Serum	Diagnostic	Increased in MBC	van Schooneveld et al., 2012
miR-299-5p	Serum	Diagnostic	Increased in MBC	van Schooneveld et al., 2012
miR-373	Plasma	Prognostic	Increased in MBC	Chen et al., 2012a,b
miR-375	Plasma	Diagnostic and prognostic	Increased in MBC; correlation to CTC status	Madhavan et al., 2012
miR-376c	Plasma	Diagnostic	Increased	Cuk et al., 2013
miR-382	Serum	Diagnostic	Increased in PBC	Mar-Aguilar et al., 2013
miR-409-3p	Plasma	Diagnostic	Increased	Cuk et al., 2013
miR-411	Serum	Diagnostic	Increased in MBC	van Schooneveld et al., 2012
miR-451	Plasma	Diagnostic	Increased in PBC	Ng et al., 2013
miR-768-3p	Plasma	Diagnostic	Decreased in MBC	Madhavan et al., 2012
miR-801	Plasma	Diagnostic	Increased	Cuk et al., 2013
miR-801	Plasma	Diagnostic and prognostic	Increased in MBC; correlation to CTC status	Madhavan et al., 2012

miRNA levels denoted as increased or decreased in cancer cases in comparison to controls unless stated otherwise. Abbreviations: CTC, circulating tumour cells; PBC, primary breast cancer; MBC, metastatic breast cancer.

**Table 3:** **Circulating miRNAs reprted to be a biomarker for lung cancer**.

**miRNA**	**Sample type**	**Type of biomarker**	**miRNA levels and clinical significance**	**References**
let-7a	Plasma and serum	Diagnostic	Increased; differences between distinct ethnic groups	Heegaard et al., 2012
miR-1	Serum	Prognostic	Decreased levels correlated to poorer OS	Hu et al., 2010
miR-15b	Plasma	Prognostic	Decreased in patients with poor outcome	Boeri et al., 2011
miR-16	Plasma	Prognostic	Decreased in aggressive disease	Boeri et al., 2011
miR-16	Serum	Diagnostic	Decreased in pre- (asymptomatic) and post-diagnostic samples	Keller et al., 2011
miR-17	Plasma	Diagnostic and prognostic	Increased; decreased in patients with poor outcome	Boeri et al., 2011
miR-17-5p	Plasma and serum	Diagnostic	Increased; differences between distinct ethnic groups	Heegaard et al., 2012
miR-19b	Plasma	Diagnostic	Increased	Boeri et al., 2011
miR-21	Plasma	Prognostic	Increased	Boeri et al., 2011
miR-21	Plasma	Diagnostic	Increased	Shen et al., 2011
miR-21	Plasma	Diagnostic and predictive	Increased; predictive for platinum-base chemotherapy.	Wei et al., 2011
miR-21	Serum	Diagnostic and prognostic	Increased; decrease in post-operative samples	Le et al., 2012
miR-21	Serum	Diagnostic and prognostic	Increased; associated with more advanced stages; correlated with OS	Liu et al., 2012a,b
miR-21	Plasma	Diagnostic	Increased in cases compared to healthy smokers	Tang et al., 2013
miR-24	Serum	Diagnostic and prognostic	Decrease in post-operative samples	Le et al., 2012
miR-25	Serum	Diagnostic	Increased	Chen et al., 2008
miR-27a	Plasma and serum	Diagnostic	Increased; differences between distinct ethnic groups	Heegaard et al., 2012
miR-28-3p	Plasma	Diagnostic and prognostic	Increased; increased in asymptomatic samples compared to controls too; increased in aggressive disease and decreased in patients with poor outcome	Boeri et al., 2011
miR-29c	Plasma and serum	Diagnostic	Decreased; differences between distinct ethnic groups	Heegaard et al., 2012
miR-30c	Plasma	Diagnostic	Increased; increased in asymptomatic samples compared to controls too	Boeri et al., 2011
miR-30d	Serum	Prognostic	Increased levels correlated to poorer OS	Hu et al., 2010
miR-30d	Serum	Diagnostic	Increased	Le et al., 2012
miR-92a	Plasma	Diagnostic	Increased; increased in asymptomatic samples compared to controls too	Boeri et al., 2011
miR-106a	Plasma	Diagnostic and prognostic	Increased; increased in aggressive disease and decreased in patients with poor outcome	Boeri et al., 2011
miR-106a	Plasma and serum	Diagnostic	Increased; differences between distinct ethnic groups	Heegaard et al., 2012
miR-126	Plasma	Prognostic	Decreased in patients with poor outcome	Boeri et al., 2011
miR-126	Plasma	Diagnostic	Decreased	Shen et al., 2011
miR-140-5p	Plasma	Diagnostic and prognostic	Increased; increased in asymptomatic samples compared to controls too; increased in aggressive disease	Boeri et al., 2011
miR-141	Serum	Diagnostic	Decreased	Liu et al., 2012a,b
miR-142-3p	Plasma	Prognostic	Decreased in patients with poor outcome	Boeri et al., 2011
miR-145	Plasma	Diagnostic	Decreased in cases compared to healthy smokers	Tang et al., 2013
miR-146b	Plasma and serum	Diagnostic	Increased; differences between distinct ethnic groups	Heegaard et al., 2012
miR-148a	Plasma	Prognostic	Decreased in patients with poor outcome	Boeri et al., 2011
miR-155	Plasma	Diagnostic, prognostic and predictive	Increased in cases and further increased in patients with metastasis; decreased in patients responding to therapy	Zheng et al., 2011
miR-155	Plasma and serum	Diagnostic	Increased; differences between distinct ethnic groups	Heegaard et al., 2012
miR-155	Plasma	Diagnostic	Increased in cases compared to healthy smokers	Tang et al., 2013
miR-182	Plasma	Diagnostic	Increased in cases and further increased in patients with metastasis; decreased in patients responding to therapy	Zheng et al., 2011
miR-182	Plasma	Diagnostic	Increased	Shen et al., 2011
miR-197	Plasma	Prognostic	Increased in aggressive disease	Boeri et al., 2011
miR-197	Plasma	Diagnostic, prognostic, and predictive	Increased in cases and further increased in patients with metastasis; decreased in patients responding to therapy	Zheng et al., 2011
miR-200c	Serum	Diagnostic	Decreased	Liu et al., 2012a,b
miR-205	Serum	Diagnostic	Increased	Le et al., 2012
miR-210	Plasma	Diagnostic	Increased	Shen et al., 2011
miR-221	Plasma	Prognostic	Increased in aggressive disease; decreased in patients with poor outcome	Boeri et al., 2011
miR-221	Plasma and serum	Diagnostic	Increased; differences between distinct ethnic groups	Heegaard et al., 2012
miR-223	Serum	Diagnostic	Increased	Chen et al., 2008
miR-451	Plasma	Diagnostic and prognostic	Increased; increased in asymptomatic samples compared to controls too	Boeri et al., 2011
miR-452^*^	Serum	Diagnostic	Decreased in pre- (asymptomatic) and post-diagnostic samples	Keller et al., 2011
miR-486	Serum	Prognostic	Increased levels correlated to poorer OS	Hu et al., 2010
miR-486-5p	Plasma	Prognostic	Increased in aggressive disease	Boeri et al., 2011
miR-486-5p	Plasma	Diagnostic	Decreased	Shen et al., 2011
miR-499	Serum	Prognostic	Decreased levels correlated to poorer OS	Hu et al., 2010
miR-518a-5p	Serum	Diagnostic	Decreased in pre- (asymptomatic) and post-diagnostic samples	Keller et al., 2011
miR-574-5p	Serum	Diagnostic	Decreased in pre- (asymptomatic) and post-diagnostic samples	Keller et al., 2011
miR-574-5p	Serum	Diagnostic	Increased	Foss et al., 2011
miR-593^*^	Serum	Diagnostic	Decreased in pre- (asymptomatic) and post-diagnostic samples	Keller et al., 2011
miR-660	Plasma	Diagnostic and prognostic	Increased; increased in asymptomatic samples compared to controls too	Boeri et al., 2011
miR-663	Serum	Diagnostic	Decreased in pre- (asymptomatic) and post-diagnostic samples	Keller et al., 2011
miR-718	Serum	Diagnostic	Decreased in pre- (asymptomatic) and post-diagnostic samples	Keller et al., 2011
miR-1228^*^	Serum	Diagnostic	Decreased in pre- (asymptomatic) and post-diagnostic samples	Keller et al., 2011
miR-1254	Serum	Diagnostic	Increased	Foss et al., 2011
miR-1972	Serum	Diagnostic	Decreased in pre- (asymptomatic) and post-diagnostic samples	Keller et al., 2011
miR-2114^*^	Serum	Diagnostic	Decreased in pre- (asymptomatic) and post-diagnostic samples	Keller et al., 2011

miRNA levels denoted as increased or decreased in cancer cases in comparison to controls unless stated otherwise. Note: The 30 miRNA signature described by Bianchi et al. (2011) has not been included here. Please refer to the original paper. Abbreviations: OS, overall survival.

**Table 4:** **Circulating miRNAs reprted to be a biomarker for colorectal cancer**.

**miRNA**	**Sample type**	**Type of biomarker**	**miRNA levels and clinical significance**	**References**
miR-15b	Plasma	Diagnostic	Increased	Giraldez et al., 2012
miR-17-3p	Plasma	Diagnostic	Increase; decrease in post-operative samples	Ng et al., 2009
miR-18a	Plasma	Diagnostic	Increased in CRC and advanced adenomas compared to controls	Giraldez et al., 2012
miR-19a	Plasma	Diagnostic	Increased	Giraldez et al., 2012
miR-21	Plasma	Diagnostic	Increased	Kanaan et al., 2012
miR-29a	Plasma	Diagnostic	Increased in CRC and advanced adenomas compared to controls	Huang et al., 2010
miR-29a	Plasma	Diagnostic	Increased	Giraldez et al., 2012
miR-29a	Serum	Diagnostic and prognostic	Increased in crc with liver metastasis compared to non-metastatic CRC	Wang and Gu, 2012
miR-92	Plasma	Diagnostic	Increase; decrease in post-operative samples; can differentiate CRC from gastric cancer IBD and controls	Ng et al., 2009
miR-92a	Plasma	Diagnostic	Increased in CRC and advanced adenomas compared to controls	Huang et al., 2010
miR-141	Plasma	Prognostic	Increased; correlated to OS	Cheng et al., 2011
miR-221	Plasma	Diagnostic and prognostic	Increased; correlated to OS and p53 score	Pu et al., 2010
miR-335	Plasma	Diagnostic	Increased	Giraldez et al., 2012
miR-601	Plasma	Diagnostic	Decreased in CRC and advanced adenomas compared to controls	Wang et al., 2012a,b,c
miR-760	Plasma	Diagnostic	Decreased in CRC and advanced adenomas compared to controls	Wang et al., 2012a,b,c
miR-1246	Plasma and serum	Diagnostic	A pseudo-microRNA representing an RNU2-1 fragment	Baraniskin et al., 2013

miRNA levels denoted as increased or decreased in cancer cases in comparison to controls unless stated otherwise. Abbreviations: CRC, colorectal cancer; IBD, inflammatory bowel disease; OS, overall survival.

**Table 5:** **Circulating miRNAs reprted to be a biomarker for gastric cancer**.

**miRNA**	**Sample type**	**Type of biomarker**	**miRNA levels and clinical significance**	**References**
let-7a	Plasma	Diagnostic	Decreased	Tsujiura et al., 2010
miR-1	Serum	Diagnostic	Increased; correlated to tumour stage	Liu et al., 2011
miR-17-5p	Plasma	Diagnostic	Increased	Tsujiura et al., 2010
miR-17-5p	Plasma	Diagnostic and prognostic	Increased; correlated to tnm staging, a poor OS	Wang et al., 2012a,b,c
miR-20a	Serum	Diagnostic	Increased; correlated to tumour stage	Liu et al., 2011
miR-20a	Plasma	Diagnostic and prognostic	Increased; correlated to tnm staging, a poor OS; independent risk predictor for prognosis	Wang et al., 2012a,b,c
miR-20a	Plasma	Diagnostic	Increased	Cai et al., 2013
miR-21	Plasma	Diagnostic	Increased; decrease in post-operative samples	Tsujiura et al., 2010
miR-21	Plasma	Diagnostic	Increased	Li et al., 2012a,b
miR-27a	Serum	Diagnostic	Increased; correlated to tumour stage	Liu et al., 2011
miR-34	Serum	Diagnostic	Increased; correlated to tumour stage	Liu et al., 2011
miR-106a	Plasma	Diagnostic	Increased; decrease in post-operative samples	Tsujiura et al., 2010
miR-106b	Plasma	Diagnostic	Increased	Tsujiura et al., 2010
miR-106b	Plasma	Diagnostic	Increased	Cai et al., 2013
miR-151-5p	Plasma	Diagnostic	Increased; decrease in post-operative samples	Li et al., 2012a,b
miR-187^*^	Serum	Diagnostic	Increased	Liu et al., 2012a,b
miR-199a-3p	Plasma	Diagnostic	Increased; decrease in post-operative samples; associated with lymph node metastasis and tnm staging	Li et al., 2012a,b
miR-218	Plasma	Diagnostic	Decreased	Li et al., 2012a,b
miR-221	Serum	Diagnostic	Increased in cancer and dysplasia compared to controls; correlated with poor differentiation of cancer; showed retrospective correlation in pre-diagnostic samples	Song et al., 2012
miR-221	Plasma	Diagnostic	Increased	Cai et al., 2013
miR-223	Plasma	Diagnostic	Increased; associated with helicobacter pylori infection	Li et al., 2012a,b
miR-371-5p	Serum	Diagnostic	Increased	Liu et al., 2012a,b
miR-376c	Serum	Diagnostic	Increased; correlated with poor differentiation of cancer; showed retrospective correlation in pre-diagnostic samples	Song et al., 2012
miR-378	Serum	Diagnostic	Increased	Liu et al., 2012a,b
miR-423-5p	Serum	Diagnostic	Increased; correlated to tumour stage	Liu et al., 2011
miR-451	Plasma	Diagnostic	Increased; decrease in post-operative samples	Konishi et al., 2012
miR-486	Plasma	Diagnostic	Increased; decrease in post-operative samples	Konishi et al., 2012
miR-744	Serum	Diagnostic	Increased; showed retrospective correlation in pre-diagnostic samples	Song et al., 2012

miRNA levels denoted as increased or decreased in cancer cases in comparison to controls unless stated otherwise. Abbreviations: OS, overall survival.

**Table 6:** **Circulating miRNAs reprted to be a biomarker for hematological cancers**.

**miRNA**	**Cancer**	**Sample type**	**Type of biomarker**	**miRNA levels and clinical significance**	**References**
let-7b	AML	Plasma	Diagnostic	Increased	Fayyad-Kazan et al., 2013
let-7d	AML	Plasma	Diagnostic	Decreased	Fayyad-Kazan et al., 2013
miR-10a-5p	AML	Serum	Diagnostic	Increased	Zhi et al., 2013
miR-15a	*de novo* DLBCL	Serum	Diagnostic	Increased	Fang et al., 2012
miR-16-1	*de novo* DLBCL	Serum	Diagnostic	Increased	Fang et al., 2012
miR-21	*de novo* DLBCL	Serum	Diagnostic	Increased	Lawrie et al., 2008
miR-29c	*de novo* DLBCL	Serum	Diagnostic	Increased	Fang et al., 2012
miR-34a	*de novo* DLBCL	Serum	Diagnostic	Increased	Fang et al., 2012
miR-92a	AML	Plasma	Diagnostic	Decreased	Tanaka et al., 2009
miR-92a	NHL	Plasma	Diagnostic and predictive	Decreased; further decreased in patients with decreased RFS	Ohyashiki et al., 2011
miR-93-5p	AML	Serum	Diagnostic	Increased	Zhi et al., 2013
miR-129-5p	AML	Serum	Diagnostic	Increased	Zhi et al., 2013
miR-150	AML	Plasma	Diagnostic and prognostic	Decreased; increased in cases with CR compared to those without CR	Fayyad-Kazan et al., 2013
miR-155	*de novo* DLBCL	Serum	Diagnostic	Increased	Lawrie et al., 2008
miR-155	*de novo* DLBCL	Serum	Diagnostic	Increased	Fang et al., 2012
miR-155-5p	*de novo* DLBCL	Serum	Diagnostic	Increased	Zhi et al., 2013
miR-181b-5p	*de novo* DLBCL	Serum	Diagnostic and prognostic	Increased; correlates to OS	Zhi et al., 2013
miR-210	*de novo* DLBCL	Serum	Diagnostic	Increased; high levels correlate with improved RFS	Lawrie et al., 2008
miR-221	*de novo* DLBCL	Plasma	Diagnostic and prognostic	Increased; correlates to OS	Guo et al., 2010
miR-320d	*de novo* DLBCL	Serum	Diagnostic	Increased	Zhi et al., 2013
miR-339	*de novo* DLBCL	Plasma	Diagnostic	Decreased	Fayyad-Kazan et al., 2013
miR-342	*de novo* DLBCL	Plasma	Diagnostic and prognostic	Decreased; increased in cases with CR compared to those without CR	Fayyad-Kazan et al., 2013
miR-523	AML	Plasma	Diagnostic	Increased	Fayyad-Kazan et al., 2013

miRNA levels denoted as increased or decreased in cancer cases in comparison to controls unless stated otherwise. Abbreviations: AML, acute myeloid leukemia; CR, complete remission; DLBCL, diffuse large B cell lymphoma; NHL, non-Hodgkin's lymphoma; OS, overall survival; RFS, relapse-free survival.

## Prostate cancer

Prostate cancer (PC) was the first cancer type to be used as a disease model for the establishment of circulating miRNAs' potential as blood-based biomarkers. The pioneer work by Mitchell and colleagues have provided very important insights and laid the foundation for future investigations on circulating miRNAs and cancers. This study demonstrated that the tumor-derived miRNAs from the donor could reach the circulation of the recipient PC xenograft mouse, and could be subsequently measured in the recipients' plasma. Additionally, Mitchell et al. also investigated a few miRNA candidates which were chosen based on their expression in human prostate specimens and absence in plasma of healthy controls. This investigation performed with serum of metastatic PC (mPC) patients and healthy control individuals found increased levels of miR-141, a miRNA involved in epithelial-mesenchymal transition, in the mPC group (Mitchell et al., 2008). In another study, pre-selected oncogenic miR-26a, miR-195, and let-7i were first confirmed to be upregulated in tissue, and then found to be significantly elevated in serum of PC patients compared to those with benign prostate hyperplasia (BPH). However, this study was not able to reproduce the significant difference in miR-141, and claimed that the miR-141 levels were too low for reliable testing (Mahn et al., 2011). To achieve the same end-result of identifying miRNAs that could discriminate PC from BPH and healthy controls, global miRNAs levels were screened for and top hits validated. A five miRNA panel consisting of let-7c, let-7e, miR-30c, miR-622, and miR-1285 was proposed (Chen et al., 2012a,b). The miRNAs described in the above studies, due to their ability to discriminate PC from even BPH, are ideal candidates for early detection of PC, especially those at high-risk.

Apart from detection, circulating miRNAs have also been connected to prognosis of PC. miR-141, miR-200b, and miR-375 were the first miRNAs identified in this context due to correlations to Gleason score and lymph node affliction (Brase et al., 2011). The prognostic promise of miR-141 and miR-375 has been confirmed by two separate studies, in which their amounts were found to increase from low-risk through high-risk localized disease toward metastatic cancer (Bryant et al., 2012; Nguyen et al., 2012). Circulating miRNAs have also been found to be linked to various PC risk scores such as tumor stage, CAPRA (Cancer of the Prostate Risk Assessment) and D'Amico scores (miR-20a, miR-21, miR-145, and miR-221) (Shen et al., 2012) or have predictive abilities similar to that of circulating tumour cell (CTC) counts, PSA, and lactate dehydrogenase (LDH) levels of PC patients (miR-21, miR-141) (Gonzales et al., 2011).

## Breast cancer

Currently, there are no early detection markers for primary breast cancer (PBC) in routine clinical use. Zhu and colleagues performed the first study to understand the association between serum miR-16, miR-145, miR-155 levels, and breast cancer risk, and found a correlation of miR-155 to progesterone receptor status (Zhu et al., 2009). In one of our studies, which was the first investigation based on genome-wide approach in PBC, miR-148b, miR-376c, miR-409-3p, and miR-801 were identified as potential early stage breast cancer biomarkers in plasma. Interestingly, miR-148b, miR-376c, and miR-409-3p were present at lower levels in malignant tissue compared to benign breast tissue, whereas no difference could be detected in miR-801 (Cuk et al., 2013). An alternate approach of identifying circulating miRNAs capable of early detection, combed for miRNAs commonly deregulated in tissue and plasma. This led to the identification of decreased miR-145 and increased miR-451 levels in plasma of breast cancer patients in relation to controls, and their plasma levels reflected those of tissue (Ng et al., 2013). Contrarily, miR-145 was reported to be upregulated in serum of PBC in another study, which could be due to the analysis of different sample types by the two studies (Mar-Aguilar et al., 2013). Recently, analysing circulation miRNAs that are known to be aberrantly expressed in breast tissue or have functional role in tumorigenesis has led to the addition of miR-21 and miR-92a (Si et al., 2013), miR-10b, miR-125b, miR-155, miR-191, and miR-382 (Mar-Aguilar et al., 2013), and miR-30a (Zeng et al., 2013) to this growing list of miRNAs for early detection of PBC. Interestingly, while exploring the difference in plasma miRNA levels between two distinct ethnic groups (Caucasian and African-American), different miRNAs were found to be deregulated in the Caucasian American and the African-American patients in comparison to their ethnically matched controls, with an overlap of only two miRNAs (miR-181a and miR-1304) between them, thus showing that circulating miRNA profile could depend on ethnicity to some degree (Zhao et al., 2010).

With respect to the metastatic setting, many reports outline the identification of circulating miRNAs which have the ability to discriminate metastatic breast cancer (MBC) from PBC and/or controls. One such study focusing on both PBC and MBC uncovered a significant difference in serum total RNA and miR-155 between PBC and healthy controls, while miR-10b, miR-34a, and miR-155 correlated with overt metastasis (Roth et al., 2010). In contrast to this candidate gene-approach, van Schooneveld et al. found miR-215, miR-299-5p, and miR-411 to be increased in tissue and also in serum of patients with MBC compared to healthy individuals by Taqman low density array cards (van Schooneveld et al., 2012). In a genome-wide array based approach, focusing not only on the identification of miRNAs for detection of MBC, but also those which could serve as prognostic markers, we investigated plasma from patients and controls, and correlated them to CTC status. We detected nine miRNAs (miR-141, miR-200a, miR-200b, miR-200c, miR-203, miR-210, miR-375, and miR-801) which were able to discriminate between MBC patients based on the presence or absence of CTCs, as well as MBC patients from healthy individuals. Combinations of these circulating miRNAs or even miR-200b alone were a better marker of progression-free survival and overall survival (OS) than CTC status, which is an FDA-approved prognostic marker for MBC (Madhavan et al., 2012). Another recent study interrogated plasma miR-10b and miR-373 levels, which are known mediators of metastasis in breast cancer, wherein they uncovered association of these miRNAs with lymph node metastasis, thereby showing potential as prognostic biomarkers (Chen et al., 2012a,b).

Apart from detection and disease prognosis, circulating miRNAs have also been associated with response to breast cancer treatment and thus possess predictive capabilities. Serum miR-125b (Wang et al., 2012a,b,c) and miR-155 (Sun et al., 2012) have been linked to chemotherapy response. Furthermore, miR-30a and miR-155 have been demonstrated as more accurate than the predictive markers CEA and CA 15-3, which are currently in use for monitoring treatment in MBC (Harris et al., 2007; Uehara et al., 2008; Sun et al., 2012; Zeng et al., 2013).

## Lung cancer

Lung cancer has been extensively studied with respect to its circulating miRNA profile and majority of studies reviewed here have concentrated on non-small-cell lung cancer (NSCLC), the more common type. The first study to systematically characterize miRNAs in serum by Solexa sequencing identified sixty three circulating miRNAs in NSCLC patients that were not detected in control samples, and ultimately miR-25 and miR-223 were highlighted as the most promising diagnostic markers (Chen et al., 2008). Another study adopting a global miRNA screening recommended miR-574-5p and miR-1254 as detection markers (Foss et al., 2011). As opposed to this global approach, Shen et al. verified miRNAs in plasma of NSCLC patients and controls, which were previously reported as differentially expressed in sputum. A panel of four miRNAs (miR-21, miR-126, miR-486-5p, and miR-210) was declared as having the highest predictive power for all stages of NSCLC, with a higher diagnostic sensitivity for more advanced stages (Shen et al., 2011). The diagnostic value of miR-21, once again, along with miR-145 and miR-155 was confirmed by validating their significant deregulation between malignant NSCLC and healthy smokers (Tang et al., 2013).

While the above studies aimed to identify circulating miRNAs that could identify NSCLC patients, Boeri et al. and Bianchi et al. attempted to distinguish miRNAs that could also be useful in the diagnosis of asymptomatic patients in plasma and serum, respectively (Bianchi et al., 2011; Boeri et al., 2011). The first study conducted a comprehensive analysis, and put forth a miRNA signature in plasma capable of detecting lung cancer in asymptomatic patients before clinical presentation of disease. miR-28-3p, miR-30c, miR-92a, miR-140-5p, miR-451, and miR-660 were the most robust miRNAs to predict the development of lung cancer, as they were deregulated in samples collected from patients even 1–2 years prior to diagnosis when compared to control individuals. In case of samples collected at the time-point of diagnosis, miR-17, miR-19b, miR-92a, miR-106a, and miR-660 were also found to be informative (Boeri et al., 2011). Bianchi and co-workers analysed serum samples from asymptomatic NSCLC patients and healthy smokers, and identified a signature of 34 miRNAs with the capacity to (1) identify early stage NSCLC patients and, (2) discriminate between benign and malignant lesions, thus detecting the onset of a malignant disease (Bianchi et al., 2011). A retrospective study, which profiled serum miRNAs in pre- and post-diagnostic NSCLC samples, and compared them to normal controls validated miR-16, miR-452^*^, miR-518a-5p, miR-574-5p, miR-593^*^, miR-663, miR-718, miR-1228^*^, miR-1972, and miR-2114^*^ to be significantly deregulated in both categories of cases, thus making them ideal markers for early detection (Keller et al., 2011). These studies concur on their inference that dramatic changes in circulating miRNA profile occur at time points closer to cancer diagnosis, thus supporting the idea that miRNAs may predict cancer development years prior to clinically symptomatic disease.

With a very poor 5-year survival rate in lung cancer patients, markers to assess prognosis or predict therapy response are equally important. When pre-diagnostic samples of patients with an unfavorable clinical outcome were investigated, two sets of miRNAs were developed to help identify patients at risk for aggressive disease and prognosticate outcome (Boeri et al., 2011). By genome-wide sequencing of serum of patients with longer and those with shorter survival, Hu et al. determined the levels of serum miR-1, miR-30d, miR-486, and miR-499 to be linked to survival in NSCLC patients. Specifically, patients having two or more of these “high-risk” miRNAs deregulated were found to have a higher probability of shortened survival compared to patients with none or just one deregulated miRNA. The four-miRNA signature was propounded as an independent predictor of OS (Hu et al., 2010). Although in this study miR-21 was one of the top three hits of sequencing, it could not be successfully validated in a larger cohort. Nevertheless, circulating miR-21 has been repeatedly reported as being deregulated in lung cancer patients and possessing prognostic value, and has specifically been associated with (1) lymph node metastasis and advanced cancer stage, (2) patient survival prognosis, (3) sensitivity to platinum-based chemotherapy, and (4) post-operative disease monitoring (Wei et al., 2011; Le et al., 2012; Liu et al., 2012a,b). A recent study found miR-142-3p to be associated with a high risk of recurrence in early-stage lung adenocarcinoma patients, and thus proposed it as a putative serum marker for risk assessment (Kaduthanam et al., 2013). With respect to the predictive value, plasma levels of miR-155, miR-182, and miR-197 which were shown to be increased in patients, and especially those with metastasis, exhibited a significant decrease in lung cancer patients who respond to chemotherapy (Zheng et al., 2011).

Heegaard et al., in addition to identifying circulating miRNAs for diagnostic purposes in NSCLC, compared plasma and serum miRNA profile, and miRNA profile of different ethnic groups. Notably, the miRNA levels in paired plasma and serum from the early stage NSCLC patients showed no significant correlation. Reduced levels of miR-17-5p, miR-27a, miR-106a, miR-146b, miR-155, miR-221, and let-7a, and increased levels of miR-29c were detected only in the serum of NSCLC patients and not in plasma (Heegaard et al., 2012). Analogous to the findings in a breast cancer study, the miRNA profiles also displayed differences between the African American and European American ethnic groups (Zhao et al., 2010; Heegaard et al., 2012).

## Colorectal cancer

In colorectal cancer (CRC) many studies have evaluated the feasibility of circulating miRNAs for detecting early stage cancer and also as prognostic/predictive marker (Pfütze et al., 2013). Ng et al. achieved this by profiling miRNAs in tissue and plasma, and short-listing miRNAs that were differentially expressed in both. Ultimately, miR-17-3p and miR-92, both belonging to the same miRNA gene cluster and classified as oncogenic, were validated as elevated in CRC plasma and CRC tissue, in comparison to their normal counterparts. The two miRNAs were also proposed as responsive to surgery as lower levels were detected in post-operative plasma compared to pre-operative plasma, thus expanding their role as biomarkers. Interestingly, miR-92 alone had the potential to discriminate CRC samples from other diseases, such as gastric cancer (GC) and inflammatory bowel disease samples, apart from controls (Ng et al., 2009). The diagnostic potential of miR-92a was further confirmed by another study in plasma, which in addition also identified miR-29a as being increased in CRC, and similar to the previous study the miRNAs showed reduction following surgery. However, this study was not able to validate miR-17-3p as it could not be measured due to very low levels in plasma. miR-29a and miR-92a could also differentiate adenomas (pre-malignant lesions) from control samples, however their sensitivity to differentiate adenomas from carcinomas was not mentioned, which might influence their diagnostic utility (Huang et al., 2010). By miRNA profiling and subsequent validation, miR-601 and miR-760 were also suggested as potential diagnostic biomarkers of adenomas and CRC by the same group. Combining miR-29a, miR-92a, and miR-760, the detection sensitivity of early stages of CRC was further improved, especially when complemented with CEA (Wang et al., 2012a,b,c). miR-29a amounts in serum has also been reported to discriminate metastatic and non-metastatic CRC patients, specifically liver metastasis (Wang and Gu, 2012). Another study which undertook a genome-wide miRNA profiling of plasma, identified miR-15b, miR-19a, miR-19b, miR-29a, and miR-335 as being able to differentiate CRC patients from healthy individuals, while miR-18a could do so also between advanced adenomas and healthy individuals (Giraldez et al., 2012). miR-21 was identified as an early detection marker, but here, rather than profiling plasma, the strategy followed was to profile CRC and normal tissue, and validate the top hits from this discovery round in plasma (Kanaan et al., 2012). miR-1246, in spite of being designated as a pseudo-miRNA, was discerned as a diagnostic marker for pancreatic ductal adenocarcinoma and CRC in serum and plasma, and predictive for early therapy response prediction of chemo- and radiation therapy due to its decline following surgical resection. The measured signal, which was discriminating cancer and control samples, was proposed to have come from the U2 small nuclear RNA (snRNA) fragment RNU2-1f (Baraniskin et al., 2013).

Although many studies have outlined the diagnostic potential of circulating miRNAs in CRC, not many have explored their prognostic potential. One such study measured oncogenic miRNAs and identified plasma miR-221 to have prognostic prowess in addition to being diagnostic for CRC. miR-221 levels significantly correlated to OS and p53 expression (Pu et al., 2010). The only other report in this direction showed miR-141 to be highly correlated to cancer stage, complemented CEA in identifying stage IV CRC, and associated with poorer OS (Cheng et al., 2011).

## Gastric cancer

GC has one of the poorest survival rates, which makes the discovery of suitable biomarkers for early detection or prognosis all the more important (Siegel et al., 2012). Based on previous findings in GC tissue samples, miR-17-5p, miR-21, miR-106a, miR-106b, and let-7a were chosen for analysis in plasma samples. While the first four were demonstrated to be present at significantly higher levels, let-7a was lower in GC patients than in healthy individuals. In addition to this, a significant drop in the levels of miR-21 and miR-106b in post-operative paired samples, and a concordance between tissue and plasma miRNAs were also observed. The authors interpreted from these results that the levels of plasma miRNAs reflected the corresponding expression level in tumour (Tsujiura et al., 2010). Unlike this study, Liu et al. carried out a global miRNA screening in GC and healthy control serum samples, independent of miRNA profile in tissue. A five miRNA signature in serum was identified to be markedly upregulated in GC patients, and within GC samples, additionally correlated to tumour stage, thereby an advanced clinical stage. Its discriminatory power was claimed to be even higher than currently established markers, CEA and CA 19-9. This was attributed to the use of multiple miRNAs which were functionally associated to various tumorigenic processes (immune response-related: miR-20a and miR-423-5p; tissue-specific: miR-1; tumour cell growth/cycle-related: miR-27a and miR-34). This, they claimed, could prove to be more comprehensive than a conventional single protein-based biomarker (Liu et al., 2011). Another study using miRNA profiling of serum uncovered 16 upregulated miRNAs in the discovery round. These included miR-17, miR-20a, miR-21, and miR-106a/b among others, which eventually could not be validated unlike in the previous studies mentioned here. After validation, miR-221, miR-376c, and miR-744 were delineated as markers for GC detection, capable of identifying GC even 5 years prior to clinical diagnosis (Song et al., 2012). By comparing pre- and post-operative plasma samples, miR-451 and miR-486 (both lowered in post-operation) were selected as candidates and confirmed to be present in higher amounts in GC as opposed to controls (Konishi et al., 2012). One more study dealing with GC plasma samples demonstrated miR-21 and miR-223 to be significantly higher in GC patients than in healthy controls, while miR-218 was significantly lower. Interestingly, miR-223 was also specifically correlated to *Helicobacter pylori* infection, an etiological agent for GC (Li et al., 2012a,b). Other miRNAs proposed as diagnostic markers for GC include miR-151-5p and miR-199a-3p (Li et al., 2012a,b), and miR-221 (Cai et al., 2013) which were identified in plasma. The latter study also re-affirmed the potential of miR-20a and miR-106b to differentiate GC from controls (Cai et al., 2013). Of these, miR-199a-3p was also associated with lymph node metastasis and TNM staging, thus increasing its diagnostic importance (Li et al., 2012a,b). With respect to serum, miR-187^*^, miR-371-5p, and miR-378 were found to be significantly elevated in GC patients, with miR-378 being singled out as the most informative (Liu et al., 2012a,b).

Of the above mentioned miRNAs, miR-17-5p, and miR-21 were tested in an independent study to not only be elevated in GC, but also significantly correlated to TNM staging, and a poor OS, and miR-21 alone was defined as an independent prognostic marker (Wang et al., 2012a,b,c). Other than this one report, not many studies focusing on the prognostic role of circulating miRNAs in GC have been published.

## Haematological cancers

Although circulating miRNAs from plasma or serum are in close contact with their cellular counterpart and could easily reflect any abnormality of blood cells, there has been very little research in this direction. The first foray into the field of circulating miRNAs in cancer was with diffuse large B cell lymphoma (DLBCL) where tumour-associated miR-21, miR-155, and miR-210 were shown to be significantly higher in the serum of cancer patient serum than in healthy controls. In addition to its diagnostic proficiency, miR-21 also showed correlation to relapse-free survival (Lawrie et al., 2008). miR-15a, miR-16-1, miR-29c, miR-34a, and miR-155, which are known tumour-associated miRNAs, were later also defined as early detection markers for DLBCL, with miR-155 being the only common miRNA in these two studies (Lawrie et al., 2008; Fang et al., 2012). Since the miR-17-92 polycistronic miRNA cluster plays a crucial role in lymphomagenesis and affects neo-angiogenesis, one study explored the diagnostic and prognostic value of plasma miR-92a in non-Hodgkin's lymphoma (NHL). miR-92a was found to be extremely low in patients, and additionally evinced a strong correlation to relapse rates among patients (Ohyashiki et al., 2011). Another study, extrapolating the functional role of miRNAs in cancer to its diagnostic potential, screened for miR-221. Based on the hypothesis that miRNAs functionally involved in resistance to a particular chemotherapeutic drug would harbor information regarding treatment response, Guo et al. measured miR-221 (increased in adriamycin resistant cancer cells) in extra-nodal natural killer T-Cell (NK/T-cell) lymphoma, which is a very aggressive form with very poor prognosis. Plasma miR-221 was able to distinguish patients from controls, and also demonstrated prognostic value by being correlated to OS (Guo et al., 2010).

Leukemia is another type of hematological cancer where circulating miRNAs have been probed. In addition to NHL, miR-92a has also been observed to be decreased in plasma of acute myeloid leukemia (AML) patients. This study reported the ratio of miR-92a/miR-638 as having the strongest detection value for AML, where miR-638 was considered an internal reference gene (Tanaka et al., 2009). As opposed to this knowledge-based approach, a recent article outlined the identification of candidate miRNAs from microarray experiments in plasma. let-7d, miR-150, miR-339, and miR-342 were identified to be depressed, whilst let-7b, and miR-523 to be increased in AML patients at diagnosis when compared to controls. The combination of miR-150 and miR-342 was found to be the most accurate model for diagnostic purposes, and their prognostic value was also contemplated due to their elevation in patients with complete remission (Fayyad-Kazan et al., 2013). Another study on AML carried out whole miRNome sequencing to identify candidate miRNAs in serum differentially regulated from controls, and was able to successfully validate six miRNAs; miR-10a-5p, miR-93-5p, miR-129-5p, miR-155-5p, miR-181b-5p, and miR-320d. More importantly, miR-181b-5p levels in serum showed significant association with OS (Zhi et al., 2013).

## Conclusion and future perspectives

We have seen a tremendous growth of interest in the area of circulating miRNAs that could be developed as cancer biomarkers. From the body of work presented here some interesting observations and interpretations can be made. A few miRNAs seem to be prevalent and are associated with different cancer, for e.g., oncogenic miR-17-92 cluster which shows involvement in both solid and blood cancers, hence their use as diagnostic markers could be a double edged sword for example, while they could cover a broad spectrum of cancers, they would lack specificity. Circulating miRNAs that can identify cancer development years prior to disease onset or in asymptomatic individuals, as shown in lung and GC, could be used for the screening of high-risk groups (Song et al., 2012). Different approaches have been adopted to identify these circulating miRNA biomarkers such as genome-wide profiling, knowledge-based candidate approach, analysing for those miRNAs known to be deregulated in tissue, etc., with each approach having its own advantages and disadvantages. From the conflicting results regarding concordance between miRNAs in tumour tissue and those in circulation between studies, one can infer that the circulating miRNAs have a heterogeneous origin, which has also been corroborated by other studies (Turchinovich et al., 2012). Finally, most studies propose combination of miRNAs rather than a single miRNA for discriminating cancer patients from controls, or for determining prognosis of patients, implying the advantage of using miRNAs involved in different physio-pathological pathways to obtain a complete picture.

Crucial to the translational value of any biomarker is its robustness and reproducibility. As with all upcoming fields, there are a few technical glitches, such as sample preparation for plasma or serum and data normalization, that still need to be dealt with. Establishing a standardized protocol which should be preferably followed across all studies is very important and desirable. This would facilitate comparison and (re-)affirmation of results from different cohorts and enhance detection of only those circulating miRNAs that give us information about the biological state of samples rather than confounding us with physiological or systemic differences. Most studies, with very few exceptions, deal with relatively small sample sizes and lack validation in independent cohort(s). To achieve true translational relevance and bring circulating miRNAs into routine diagnostics, we need to invest in larger study cohorts and reproducibly validate the results. Nevertheless, all the current data underlines the enormous prospective for circulating miRNAs in the field of cancer as (1) diagnostic biomarkers for early detection of cancer; (2) prognostic biomarkers helping to predict outcome and (3) predictive biomarkers which are associated with response or resistance to a particular therapy option, and thus aid in treatment planning.

### Conflict of interest statement

The authors declare that the research was conducted in the absence of any commercial or financial relationships that could be construed as a potential conflict of interest.
